# Supplementary material for: Supramolecular dynamics-enhanced synergistic antifouling mechanisms for enhanced membrane antifouling and permeability
Source: Nat Commun. 2025 Jul 30;16:6956. doi: 10.1038/s41467-025-62231-w (PMC12311053; doi:10.1038/s41467-025-62231-w)
Supplement: Supplementary file 1 — Supplementary Information [file 41467_2025_62231_MOESM1_ESM.pdf]

Supplementary Information

Supramolecular Dynamics-Enhanced Synergistic Antifouling Mechanisms for Enhanced Membrane Antifouling and Permeability

Mingrui He[1], Yulun He[1], Dongwei Lu[1*], Mengfei Wang[1], Junjie Yang[1], Tong Wu[1] and Jun Ma[1*]

[1]State Key Laboratory of Urban-Rural Water Resource & Environment, School of Environment, Harbin Institute of Technology, Harbin 150090, PR China.

[*]E-mail: lvdongwei126@126.com, majun@hit.edu.cn

This file includes:

1. Materials and Methods

2. Supplementary Fig. 1 to 13

3. Supplementary Table 1 to 12

4. References

1 Supplementary Methods

1.1 Materials and reagents

Aminopropyl terminated polydimethylsiloxane (PDMS, Mn = 5,000 Da, 99%; containing average 66 dimethyl siloxane units) was supplied by Beijing InnoChem Science & Technology Co., Ltd. (China). Cyclodextrins (α-CD, β-CD, γ-CD, ≥98%), glycidyl methacrylate (GMA, 97%, MEHQ stabilized), fluorescein-4-isothiocyanate (FITC, 99%), polyvinylpyrrolidone (PVP, Mw = 10,000 Da), bovine serum albumin (BSA, ≥96%), sodium dodecyl sulfate (SDS, ≥98%), sodium alginate (SA, AR), fulvic acid (FA, ≥90%), sodium hypochlorite solution (AR, 6-14% active chlorine basis) and oxalic acid (AR) were obtained from Shanghai Aladdin Biochemical Technology Co., Ltd. (China). Polyethersulfone (PES, Ultrason® 6020 P, Mw = 29,000 Da) was procured from BASF SE (Germany) and vacuum-dried at 70°C for 24 h before use. Pluronic® F127 was sourced from Sigma-Aldrich (Germany). Cyclohexane (AR), hexadecane (AR), and dimethylformamide (DMF, AR) were purchased from Shanghai Macklin Biochemical Co., Ltd. (China). Sodium hydroxide (NaOH, AR), hydrochloric acid (HCl, AR), and sodium chloride (NaCl, AR) were acquired from Tianjin Guangfu Technology Development Co., Ltd. and Kemiou Chemical Reagent Co., Ltd. (China). Lyophilized yeast powder was acquired from Shanxi HuaTai Biotechnology Co., Ltd. (China). Ultrapure water (18.2 MΩ·cm) was generated using an Elix® Essential 5 water purification system (Merck Millipore, USA). All chemicals were used as received without further purification.

1.2 Characterization

1.2.1 Nuclear magnetic resonance spectroscopy (NMR)

Liquid-state ^{1}H NMR Spectra were performed on a Bruker DRX 500 Spectrometer (Germany), operating at a 600 MHz at 30°C. Prior to analysis, the samples were dried in a hot air oven at 80°C for 24 hours, and subsequently dissolved in DMSO-d6.

1.2.2 Field emission scanning electron microscopy (FESEM)

The surface morphologies of the membranes were observed using a ZEISS Sigma500 FESEM (Germany) at an accelerating voltage of 10 kV. Samples were freeze-dried and coated with a thin layer of platinum via sputtering prior to imaging.

1.2.3 Fluorescence microscopy

Fluorescence characterization was performed using a Shanghai Caikon FCK-50C fluorescence microscope (China), equipped with a 100W mercury lamp UV source and operated in reflective fluorescence mode. The aperture and field diaphragms were kept fixed across multiple measurement sets.

1.2.4 X-ray photoelectron spectroscopy (XPS)

The surface chemical compositions of the membranes were quantitatively analyzed using a Thermo Scientific ESCALAB 250 XPS (USA) with Monochromated Mg Ka 150 W as the radiation source. The photoelectron take-off angle was set at 90º.

Calibration was performed using reference materials, including PES, PDMS, and CD, to adjust the sensitivity factors for the detected elements (C, O, Si, and S), ensuring accuracy in the quantitative analysis.

1.2.5 Contact angle

Contact angle measurements on the membrane surface were performed by a Jinshengxin QSPJ-360 contact angle goniometer (China) at 25ºC. Prior to measurement, the membrane surfaces were freeze-dried. The advancing contact angle (θ_a) and receding contact angle (θ_r) were measured by adding or removing approximately 5 μL of liquid from pre-formed 15 μL water droplets using a microsyringe. The surface free energy (γ) was calculated based on the three-liquid Lifshitz-van der Waals acid-base model.

$$\gamma_i = \gamma_i^{LW} + 2\sqrt{\gamma_i^+ \gamma_i^-} \quad (1)$$

$$\gamma_L(1 + \cos\theta) = 2(\sqrt{\gamma_S^{LW}\gamma_L^{LW}} + \sqrt{\gamma_L^+ \gamma_S^-} + \sqrt{\gamma_S^+ \gamma_L^-}) \quad (2)$$

where θ is the Young's contact angle, i denotes either the solid (S) or the liquid (L) phase, and γ_i^{LW}, γ_i^+ and γ_i^- (mJ/m^2) are the Lifshitz-van der Waals, acid and base components, respectively. Two polar liquids (water and glycerol) and one apolar liquid (diiodomethane) were selected as test liquids.

1.2.6 Surface pore size measurement

Surface pore size distribution was measured using a Shenqian POROLIQ 1000 liquid-liquid displacement pore size analyzer (China). The membrane was first fully wetted with water, which acted as the wetting phase. Subsequently, n-butanol was used to displace the water, and the pore size distribution was determined based on the displacement pressure required. Measurements were performed at room temperature.

2 Supplementary Figures and Tables

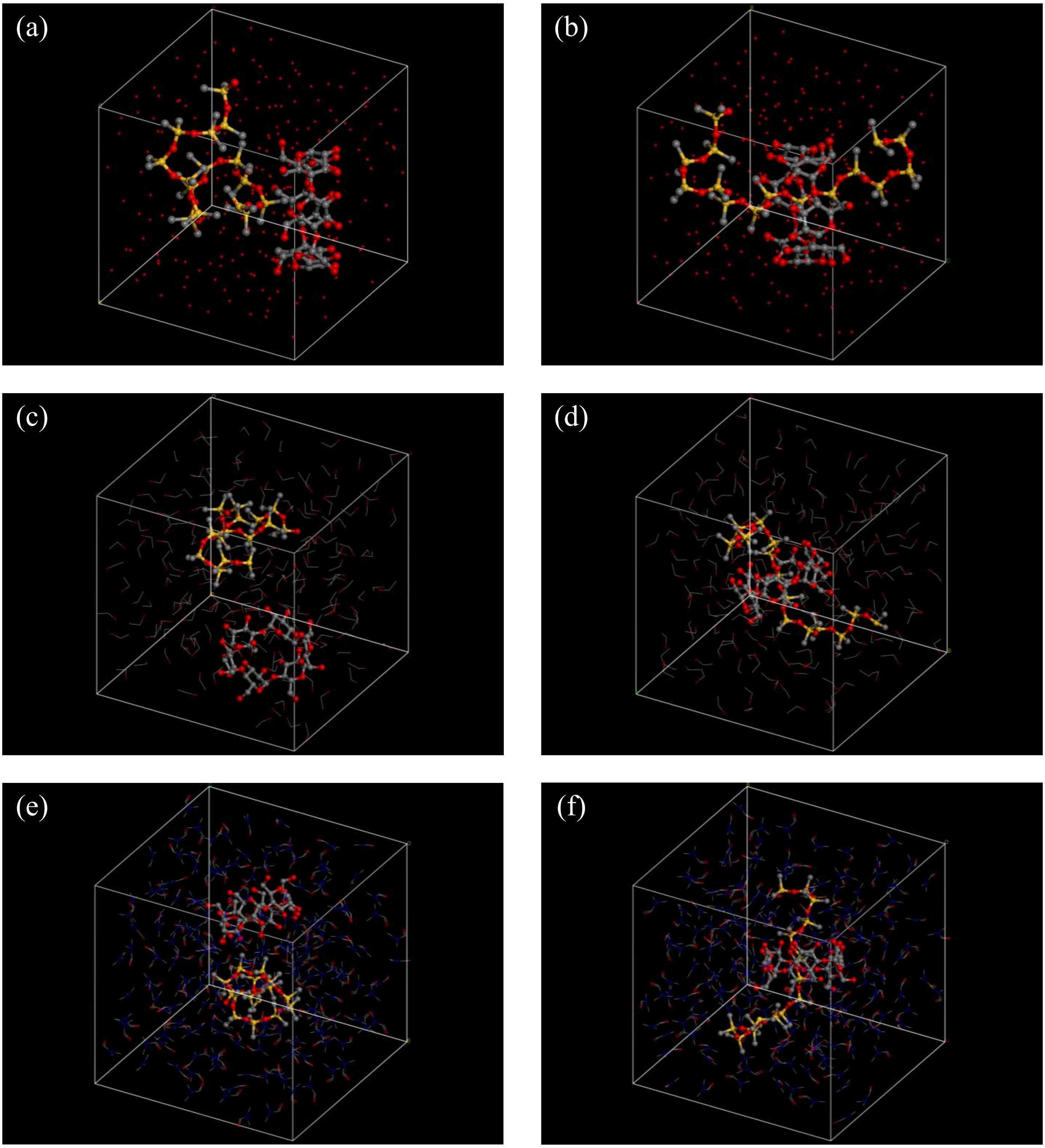

Supplementary Fig. 1 Molecular dynamics simulation of (a) unassembled α-CD/PDMS in water, (b) assembled α-CD/PDMS in water, (c) unassembled α-CD/PDMS in ethanol, (d) assembled α-CD/PDMS in ethanol, (e) unassembled α-CD/PDMS in DMF, and (f) assembled α-CD/PDMS in DMF.

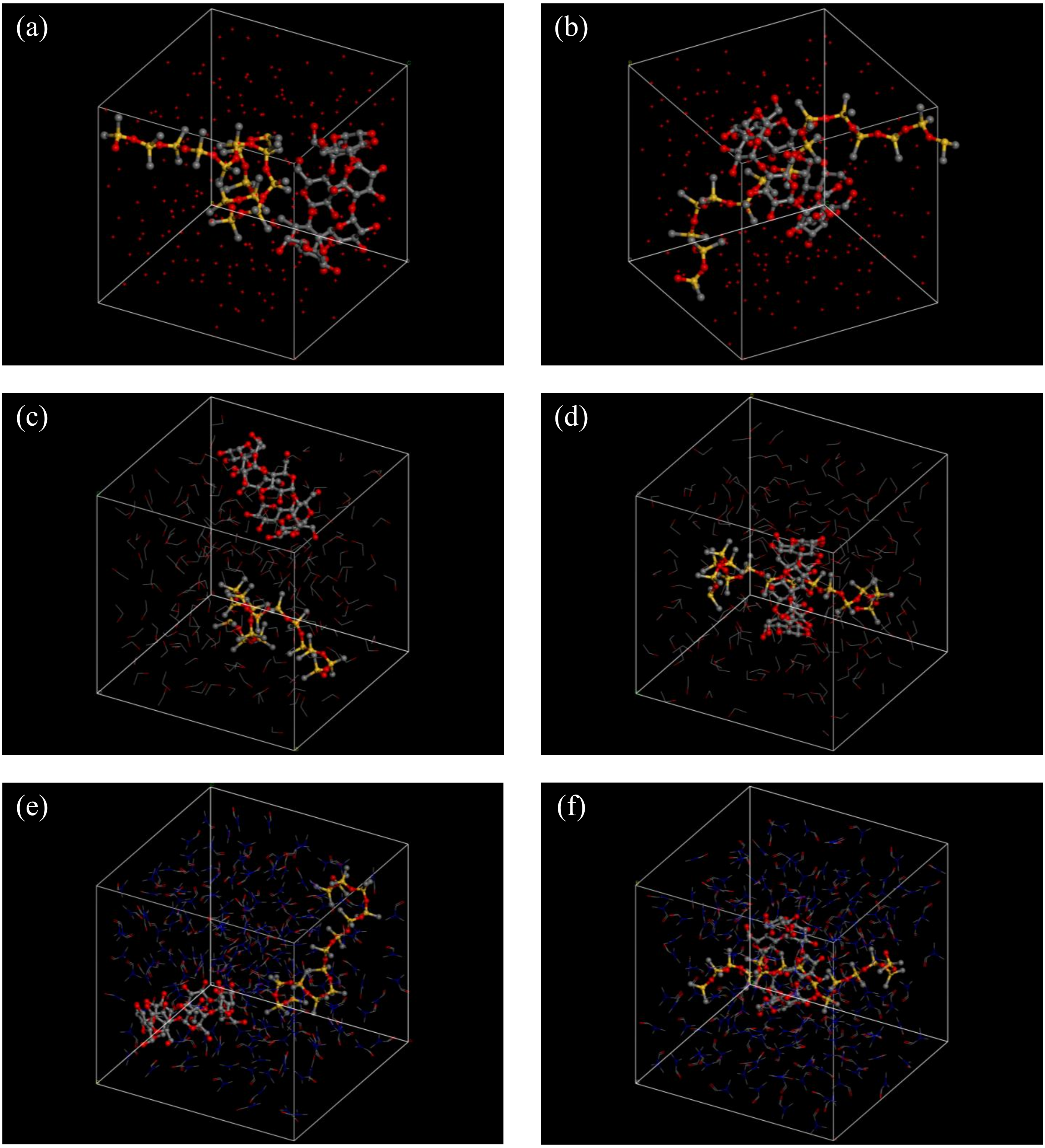

Supplementary Fig. 2 Molecular dynamics simulation of (a) unassembled β-CD/PDMS in water, (b) assembled β-CD/PDMS in water, (c) unassembled β-CD/PDMS in ethanol, (d) assembled β-CD/PDMS in ethanol, (e) unassembled β-CD/PDMS in DMF, and (f) assembled β-CD/PDMS in DMF.

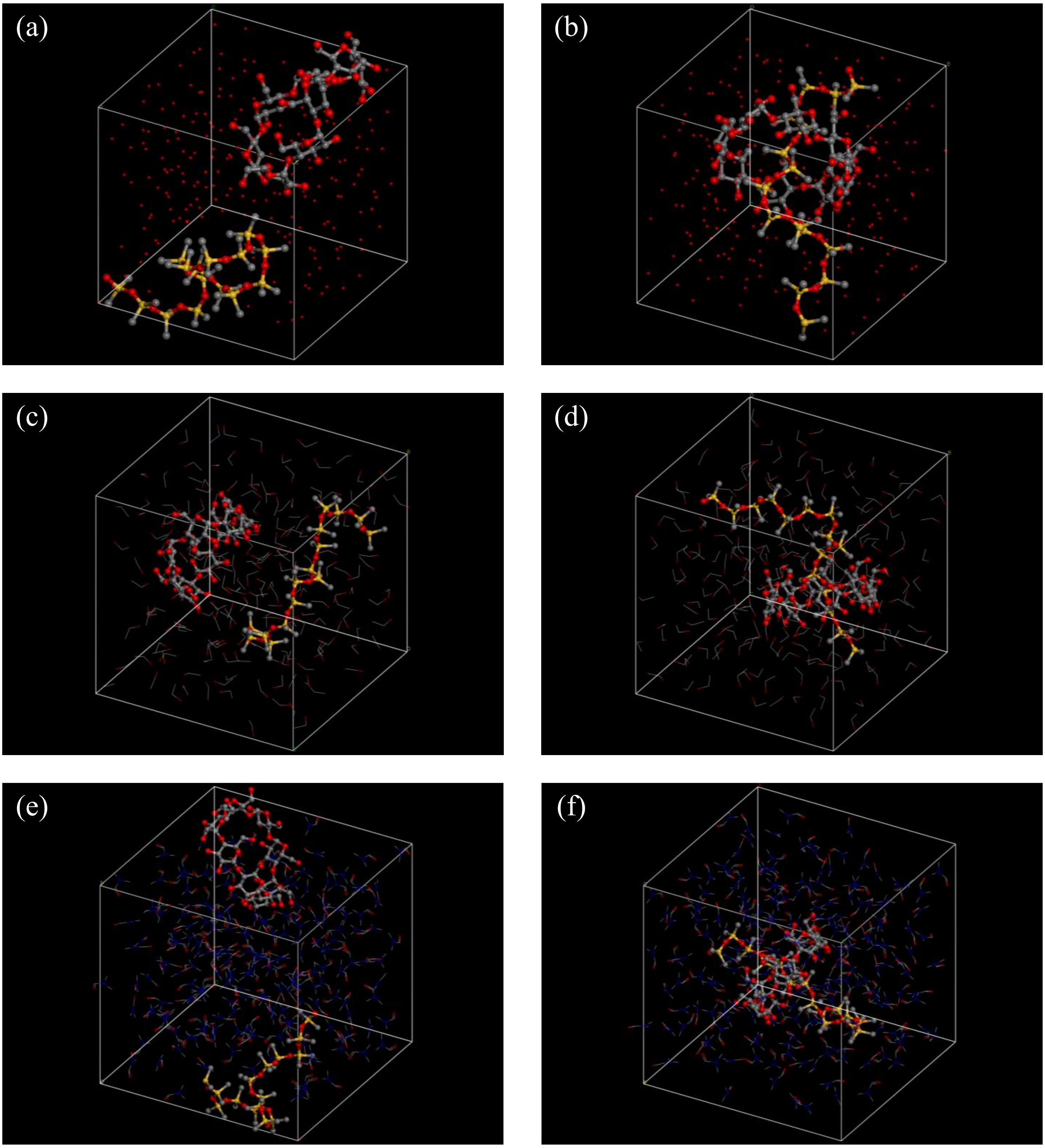

Supplementary Fig. 3 Molecular dynamics simulation of (a) unassembled γ-CD/PDMS in water, (b) assembled γ-CD/PDMS in water, (c) unassembled γ-CD/PDMS in ethanol, (d) assembled γ-CD/PDMS in ethanol, (e) unassembled γ-CD/PDMS in DMF, and (f) assembled γ-CD/PDMS in DMF.

Supplementary Table 1 System Gibbs free energy changes (ΔG) before and after the formation of CD/PDMS PPR[a]

System energy	System Gibbs free energy in different solvent environment (kcal/mol)		
	Water	Ethanol	DMF
$G_{unassembled\ \alpha\text{-CD/PDMS}}$	-1794.348	-1461.613	3041.476
$G_{unassembled\ \beta\text{-CD/PDMS}}$	-1740.327	-1412.627	3096.61
$G_{unassembled\ \gamma\text{-CD/PDMS}}$	-1677.861	-1368.187	3173.466
$G_{assembled\ \alpha\text{-CD/PDMS}}$	-1795.602	-1463.062	3037.176
$G_{assembled\ \beta\text{-CD/PDMS}}$	-1751.975	-1438.951	3075.396
$G_{assembled\ \gamma\text{-CD/PDMS}}$	-1693.745	-1399.813	3137.223
$\Delta G_{\alpha\text{-CD/PDMS assembly}}$	-1.254	-1.449	-4.3
$\Delta G_{\beta\text{-CD/PDMS assembly}}$	-11.648	-26.324	-21.214
$\Delta G_{\gamma\text{-CD/PDMS assembly}}$	-15.884	-31.626	-36.243

[a] The system included three components: CD, PDMS, and solvent (water, DMF, or ethanol).

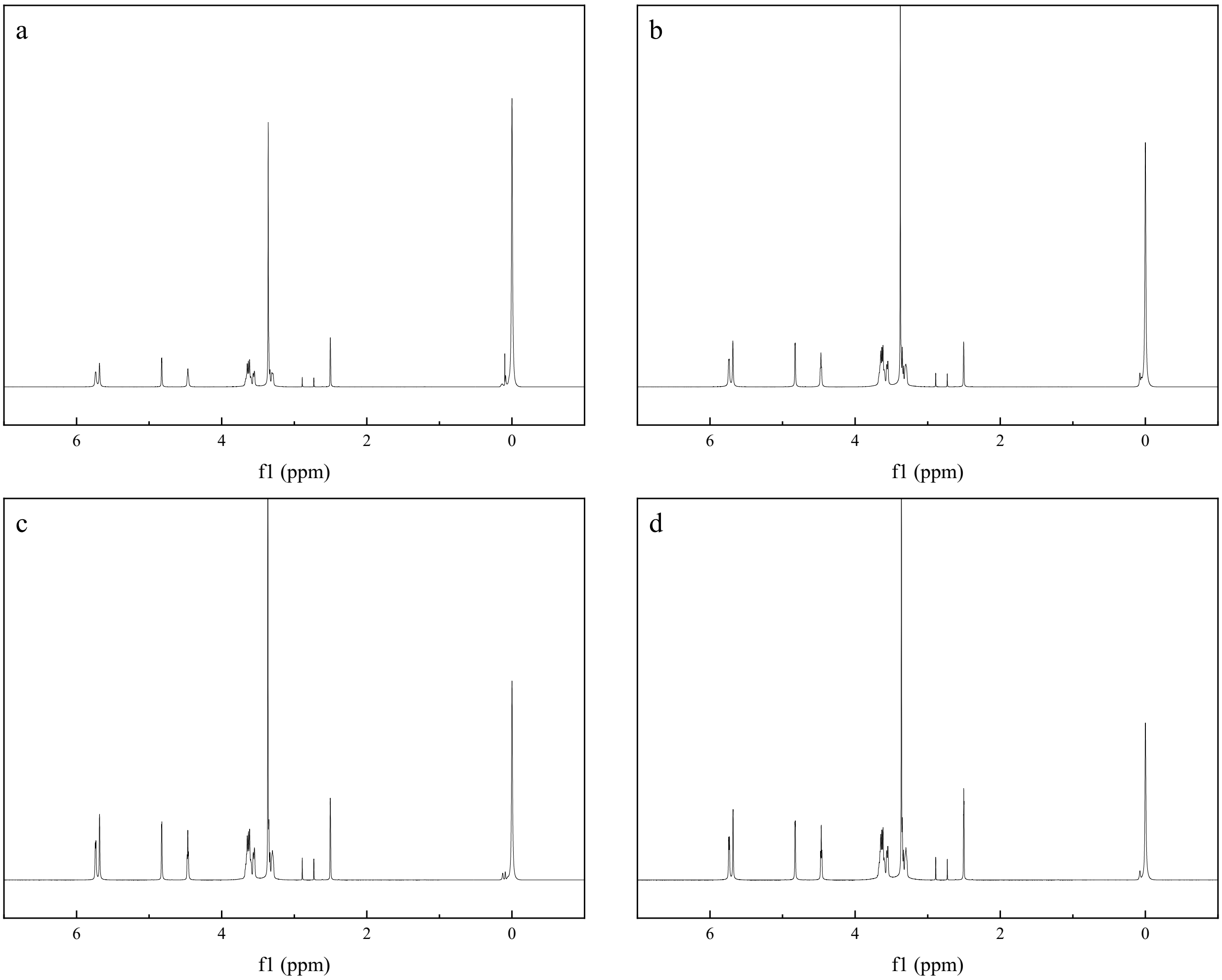

Supplementary Fig. 4 ^{1}H NMR Spectrum of β-CD/PDMS PPRs, recorded in DMSO-d_6 at 600 MHz. δ 5.70 (d, 7H, C_1H of β-CD), 4.75 (t, 7H, C_3H of β-CD), 3.63 (m, 21H, C_5H and C_6H of β-CD), 3.36 (s, H of water), 3.33 (m, 7H, C_2H of β-CD, partially obscured by water peak), 3.30 (t, 7H, C_4H of β-CD, partially obscured by water peak), 2.81 (d, methyl proton of DMF), 2.57 (m, methyl proton of dimethyl formamide, DMSO), 0.00 (m, 6H, methyl H of PDMS units). Actual $r_{CD:PDMS}$ was determined from the intensity ratio of peaks at 5.70 ppm (C_1H of β-CD) and 0.00 ppm (methyl proton of PDMS). Panels (a) to (d) represent the spectra at actual $r_{CD:PDMS}$ of 0.133, 0.273, 0.406, and 0.529, respectively.

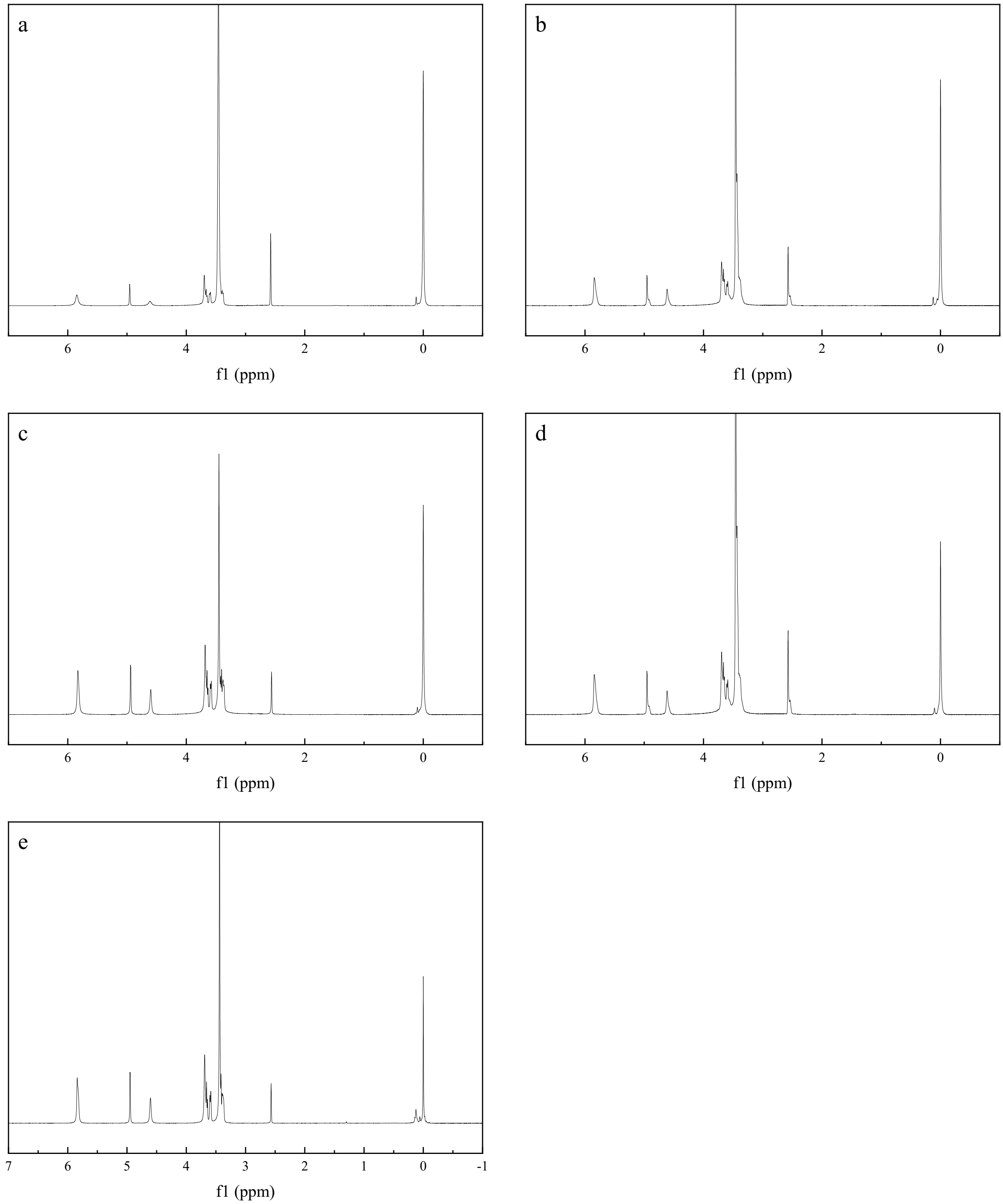

Supplementary Fig. 5 ^{1}H NMR Spectrum of γ-CD/PDMS PPRs, recorded in DMSO-d_6 at 600 MHz. δ 5.83 (d, 8H, C_1H of γ-CD), 4.77 (t, 8H, C_3H of γ-CD), 3.67 (m, 24H, C_5H and C_6H of γ-CD), 3.46 (s, H of water), 3.42 (m, 8H, C_2H of γ-CD, partially obscured by water peak), 3.39 (t, 8H, C_4H of γ-CD, partially obscured by water peak), 2.57 (m, methyl proton of DMSO), 0.0 (m, 6H, methyl H of PDMS units). Actual $r_{CD:PDMS}$ was determined from the intensity ratio of peaks at 5.83 ppm (C_1H of γ-CD) and 0.00 ppm (methyl proton of PDMS). Panels (a) to (e) represent the spectra at actual $r_{CD:PDMS}$ of 0.131, 0.270, 0.397, 0.533 and 0.674, respectively.

Supplementary Table 2 Yield and actual $r_{CD:PDMS}$ of CD/PDMS PPRs.

CD type	CD additive amount (mg)	PDMS additive amount (mg)	Yield(%)[a]	Actual $r_{CD:PDMS}$
β	590	263.3	58.5	0.133
β	590	122.5	55.9	0.273
β	590	80.6	52.3	0.406
β	590	58.4	48.8	0.529
γ	1842	751.7	83.3	0.131
γ	1842	349.2	82.4	0.270
γ	1842	229.7	81.7	0.397
γ	1842	170.4	79.7	0.533
γ	1842	133.7	77.0	0.674

[a] The yield calculated based on the quantity of CD introduced into the PPRs, discounting trace residual solvents.

Supplementary Table 3 Designation of resultant membranes.

Serial number	CD type	$r_{CD:PDMS}$	Membrane name
0	/	0	PDMS@M
1	β	0.13	β-CD/PDMS@M-0.13
2	β	0.27	β-CD/PDMS@M-0.27
3	β	0.40	β-CD/PDMS@M-0.40
4	β	0.53	β-CD/PDMS@M-0.53
5	γ	0.13	γ-CD/PDMS@M-0.13
6	γ	0.27	γ-CD/PDMS@M-0.27
7	γ	0.40	γ-CD/PDMS@M-0.40
8	γ	0.53	γ-CD/PDMS@M-0.53
9	γ	0.67	γ-CD/PDMS@M-0.67

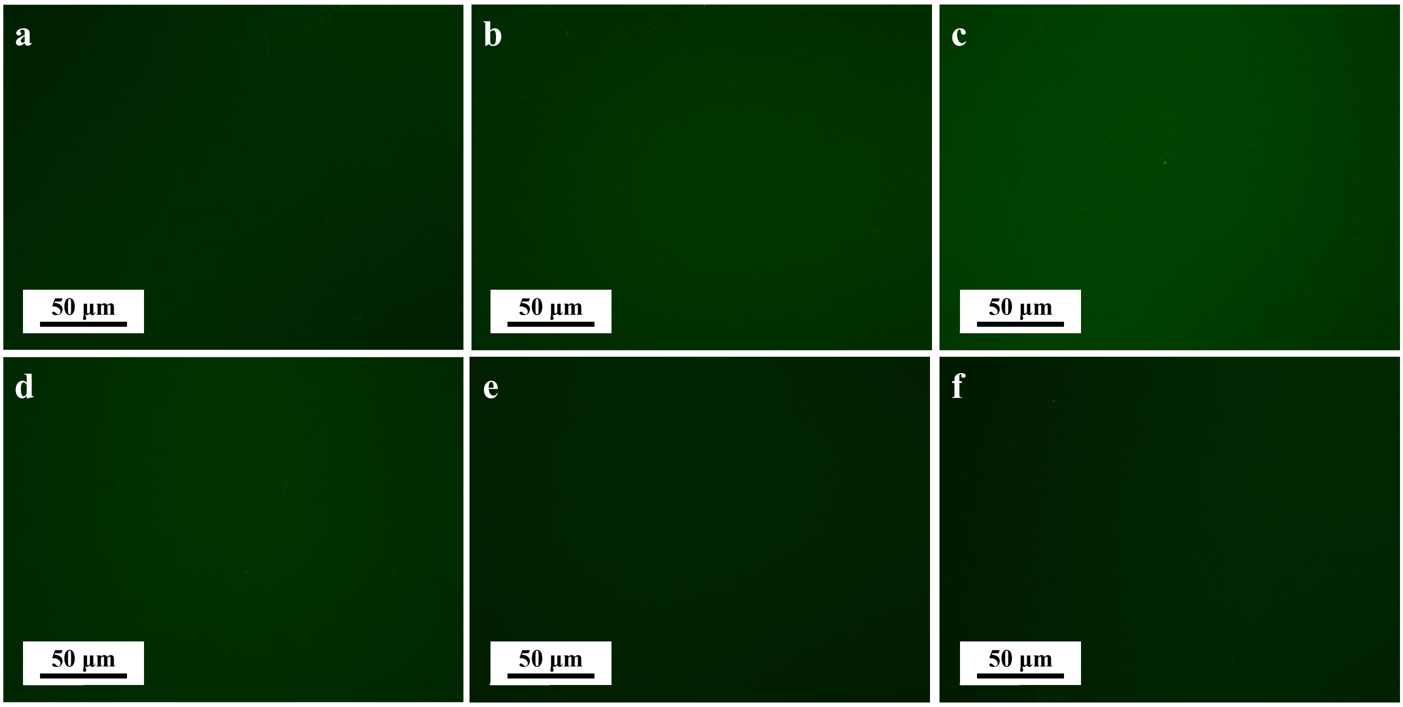

Supplementary Fig. 6 Fluorescence images of γ-CD/PDMS@M surfaces after grafting γ-CD/PDMS PPR onto membrane surface for (a) 1 h, (b) 2 h, (c) 4 h, (d) 6 h, (e) 8 h and (f) 16 h and capping with FITC for 2 h.

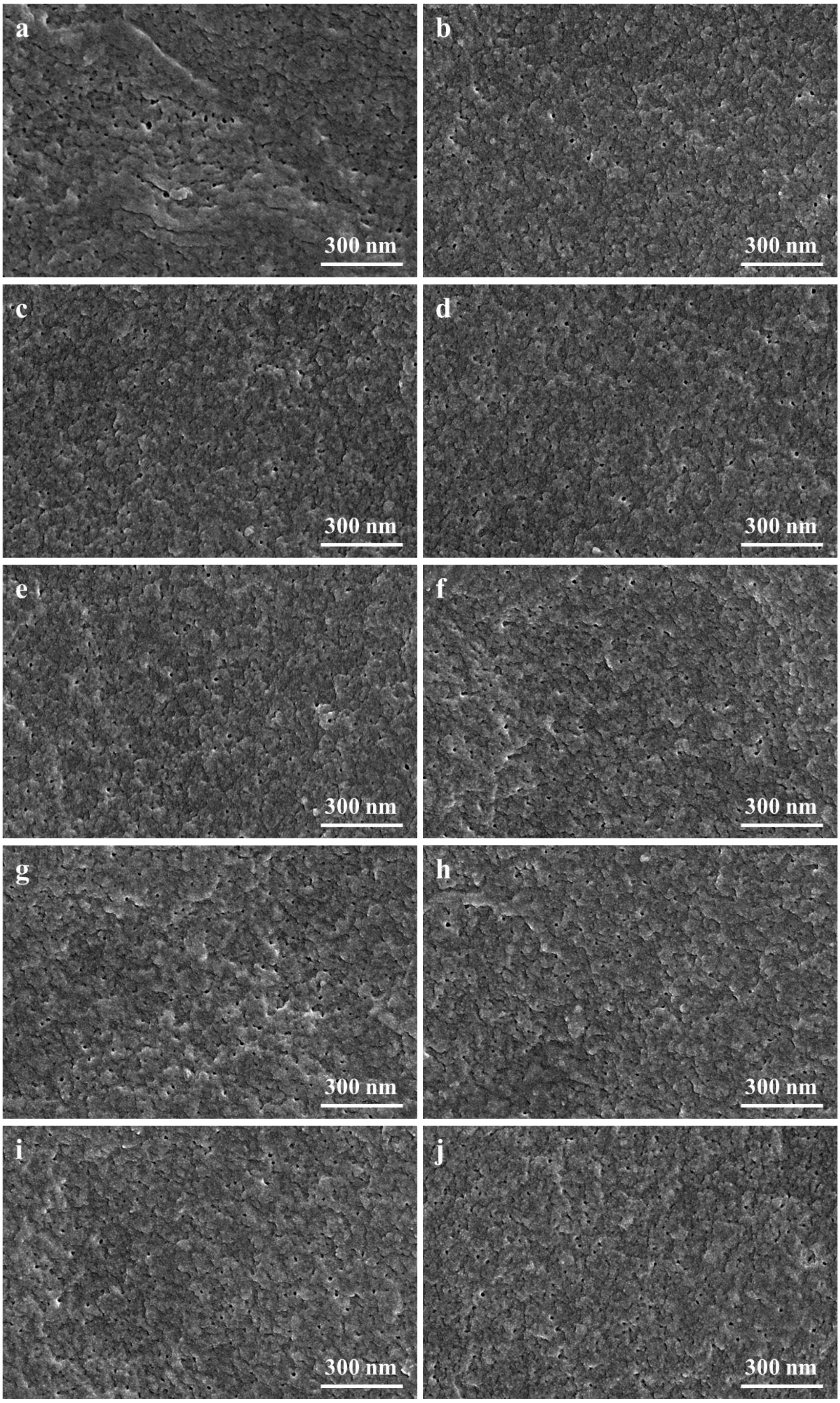

Supplementary Fig. 7 Surface SEM images of (a) PDMS@M, (b) β-CD/PDMS@M-0.13, (c) β-CD/PDMS@M-0.27, (d) β-CD/PDMS@M-0.40, (e) β-CD/PDMS@M-0.53, (f) γ-CD/PDMS@M-0.13, (g) γ-CD/PDMS@M-0.27, (h) γ-CD/PDMS@M-0.40, (i) γ-CD/PDMS@M-0.53 and (j) γ-CD/PDMS@M-0.67.

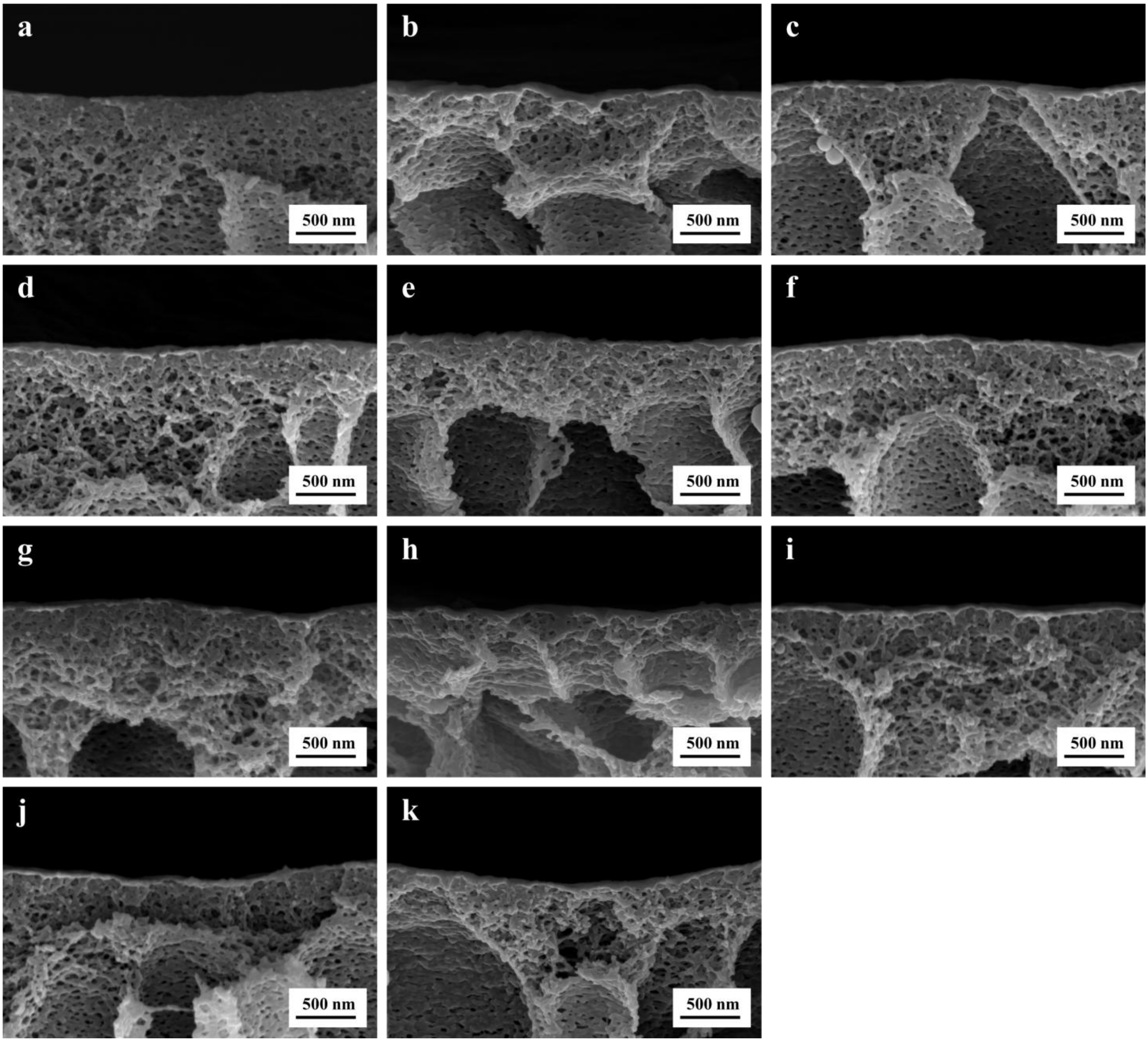

Supplementary Fig. 8 Cross-sectional SEM images of (a) BM, (b) PDMS@M, (c) β-CD/PDMS@M-0.13, (d) β-CD/PDMS@M-0.27, (e) β-CD/PDMS@M-0.40, (f) β-CD/PDMS@M-0.53, (g) γ-CD/PDMS@M-0.13, (h) γ-CD/PDMS@M-0.27, (i) γ-CD/PDMS@M-0.40, (j) γ-CD/PDMS@M-0.53, (k) γ-CD/PDMS@M-0.67.

Supplementary Table 4 Elemental composition of β-CD/PDMS@M surface[a]

$r_{CD:PDMS}$	Elemental composition (wt%)			
	C	O	Si	S
0	51.53	22.78	18.04	7.65
	51.69	22.81	17.80	7.70
0.13	55.02	30.73	6.68	7.57
	55.48	30.21	6.31	8.00
0.27	54.12	35.97	3.58	6.33
	54.38	34.44	4.44	6.74
0.4	54.14	36.52	3.17	6.17
	53.99	36.55	3.31	6.15
0.53	53.79	37.95	2.64	5.63
	54.43	36.53	2.76	6.28

[a] The elemental composition of the membrane surface was determined by integral analysis of the XPS full spectrum. Each membrane sample was tested twice starting from its preparation phase.

Supplementary Table 5 Elemental composition of the γ-CD/PDMS@M surface[a]

$r_{CD:PDMS}$	Elemental composition (wt%)			
	C	O	Si	S
0	51.53	22.78	18.04	7.65
	51.69	22.81	17.80	7.70
0.13	55.32	31.62	5.36	7.70
	55.27	31.19	5.77	7.77
0.27	54.24	34.29	4.70	6.77
	54.73	35.22	3.30	6.75
0.4	55.59	34.52	2.59	7.30
	55.26	34.86	2.80	7.08
0.53	54.10	37.30	2.23	6.37
	54.68	36.72	2.18	6.42
0.67	53.05	39.90	1.98	5.07
	52.96	39.98	2.00	5.06

[a] The elemental composition of the membrane surface was determined by integral analysis of the XPS full spectrum. Each membrane sample was tested twice starting from its preparation phase.

Supplementary Table 6 Molecular coverage of β-CD/PDMS@M surface[a]

$r_{CD:PDMS}$	Molecular coverage (wt%)			
	PES	PGMA	PDMS	β-CD
0	51.98	3.02	45.00	0.00
	52.41	3.17	44.42	0.00
0.13	52.08	3.63	16.75	27.54
	55.11	3.23	15.84	25.82
0.27	43.53	2.30	8.97	45.20
	46.36	2.47	11.13	40.04
0.4	42.42	2.56	7.94	47.08
	42.28	2.28	8.29	47.15
0.53	38.53	2.54	6.67	52.26
	43.19	2.68	6.92	47.21

[a] The molecular coverage on the membrane surface was approximated by combining the elemental composition of the membrane surface with that of the molecules themselves.

Supplementary Table 7 Molecular coverage of the γ-CD/PDMS@M surface[a]

$r_{CD:PDMS}$	Molecular coverage (wt%)			
	PES	PGMA	PDMS	γ-CD
0	51.98	3.02	45.00	0.00
	52.41	3.17	44.42	0.00
0.13	53.04	2.91	13.45	30.60
	53.51	2.90	14.48	29.11
0.27	46.56	2.17	11.78	39.49
	46.46	2.47	8.28	42.79
0.4	50.31	2.61	6.51	40.57
	48.77	2.54	7.03	41.66
0.53	41.32	1.86	5.98	50.84
	44.18	2.44	5.47	47.91
0.67	34.80	1.88	4.95	58.37
	34.29	2.46	5.01	58.24

[a] The molecular coverage on the membrane surface was approximated by combining the elemental composition of the membrane surface with that of the molecules themselves.

Supplementary Table 8 Surface energy calculation of β-CD/PDMS@M[a]

$r_{CD:PDMS}$	Test 1			Test 2			Test 3			Average	Standard deviation
	R_S	R_S^D	R_S^P	R_S	R_S^D	R_S^P	R_S	R_S^D	R_S^P		
0	31.2	27.3	3.9	31.3	27.4	3.9	30.5	26.6	3.9	31.00	0.36
0.13	32.5	27.4	5.1	32.3	27.3	5	32	26.9	5.1	32.27	0.21
0.27	34.6	28.2	6.4	33.7	27.1	6.6	33.1	26.7	6.4	33.80	0.62
0.4	36.9	27.6	9.3	35.9	27	8.9	35.7	26.7	9	36.17	0.52
0.53	39.4	27.1	12.3	39.1	26.7	12.4	39.8	27.4	12.4	39.43	0.29

[a] The units of surface energy and its components in the table are all mJ/m^2.

Supplementary Table 9 Surface energy calculation of γ-CD/PDMS@M[a]

$r_{CD:PDMS}$	Test 1			Test 2			Test 3			Average	Standard deviation
	R_S	R_S^D	R_S^P	R_S	R_S^D	R_S^P	R_S	R_S^D	R_S^P		
0	31.2	27.3	3.9	31.3	27.4	3.9	30.5	26.6	3.9	31.00	0.36
0.13	32.2	26.7	5.5	32.2	26.8	5.4	33	27.7	5.3	32.47	0.38
0.27	33.7	26.3	7.4	33.9	26.7	7.2	34.7	27.4	7.3	34.10	0.43
0.4	36.2	26.7	9.5	36.9	27.2	9.7	36.8	27.2	9.6	36.63	0.31
0.53	40.9	27.3	13.6	40.3	27.1	13.2	41	27.8	13.2	40.73	0.31
0.67	49.4	27.8	21.6	49.9	27.5	22.4	50.3	27.6	22.7	49.87	0.37

[a] The units of surface energy and its components in the table are all mJ/m^2.

Supplementary Table 10 Surface energy calculation of BM[a]

Test 1			Test 2			Test 3			Average	Standard deviation
R_S	R_S^D	R_S^P	R_S	R_S^D	R_S^P	R_S	R_S^D	R_S^P		
39.3	37.3	2	37.4	35.2	2.2	37.1	35	2.1	37.93	0.97

[a] The units of surface energy and its components in the table are all mJ/m^2.

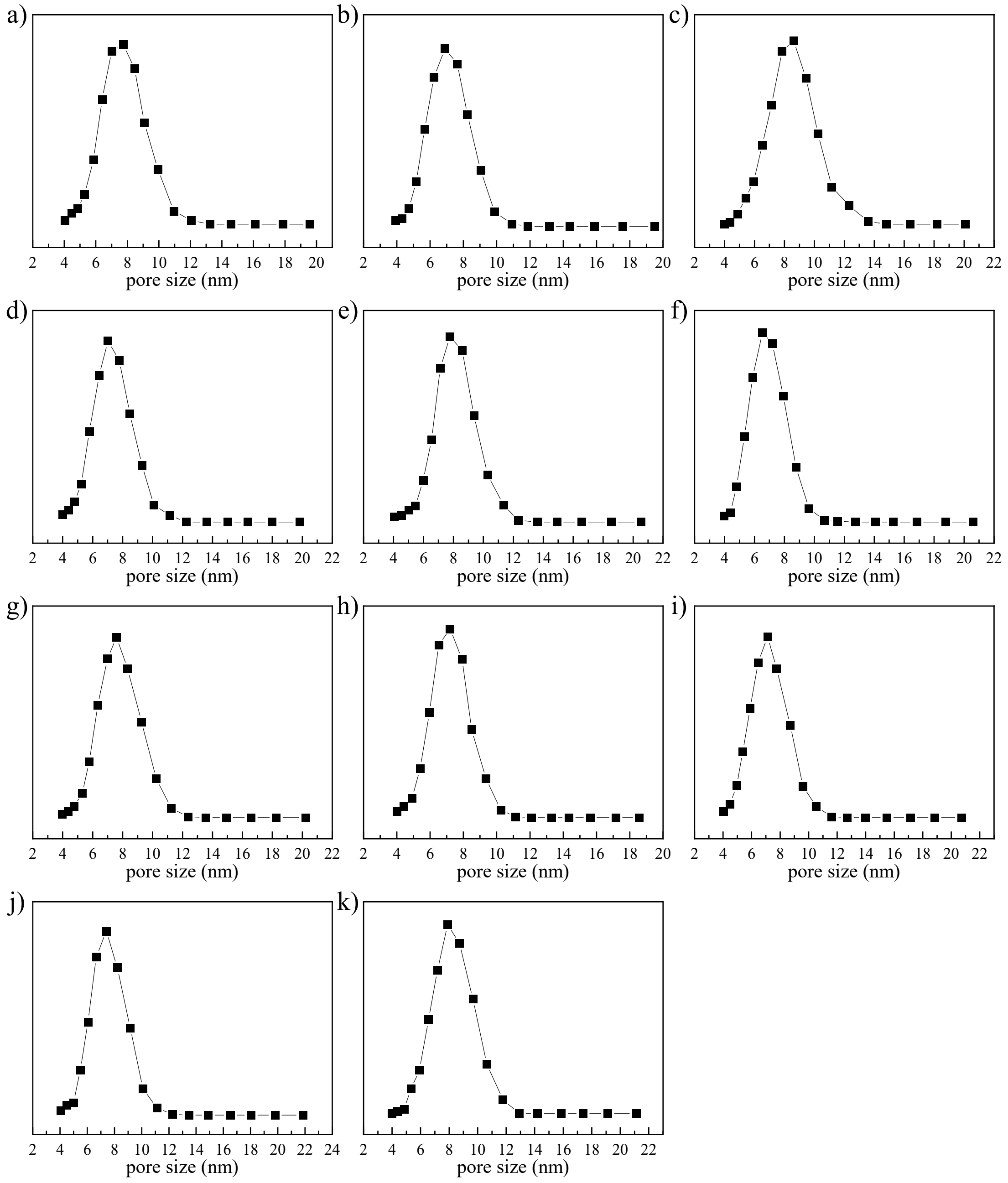

Supplementary Fig. 9 Pore size distribution of (a) BM, (b) PDMS@M, (c) β-CD/PDMS@M-0.13, (d) β-CD/PDMS@M-0.27, (e) β-CD/PDMS@M-0.40, (f) β-CD/PDMS@M-0.53, (g) γ-CD/PDMS@M-0.13, (h) γ-CD/PDMS@M-0.27, (i) γ-CD/PDMS@M-0.40, (j) γ-CD/PDMS@M-0.53, (k) γ-CD/PDMS@M-0.67, measured via liquid-liquid displacement method.

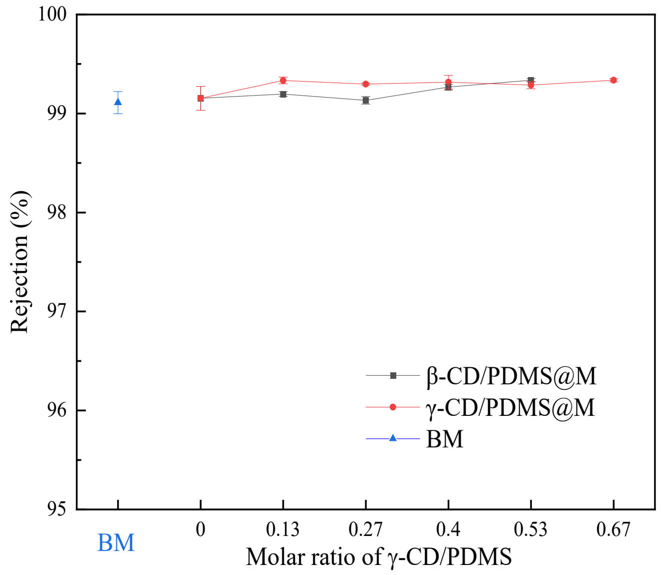

Supplementary Fig. 10 BSA rejection rate of CD/PDMS@Ms. Data are presented as mean ± SD (n = 3).

Supplementary Table 11 Comparison of reported hydrophilic-LSE heterogeneous membrane performances

Report date	Hydrophilic substances	LSE substances	Membrane permeability ($Lm^{-2}h^{-1}bar^{-1}$)	Model foulants	Fouling duration, stirring speed (rpm), flux decline rate (FDR)	Cleaning methods, flux recovery rate (FRR)	Variation of antifouling performance at different tangential flow or different microdomains
2011[1]	PEGMA	HFMB	75	Vacuum pump oil/water emulsion	60 min, 300, 3.4%	Deionized water surface washing, 100%	FDR of 40% and FRR of 70% at the stirring speed of 0 rpm
2013[2]	PEG	Siloxane	166.7	BSA	3h, /, 73%	Deionized water surface washing, 84.9	BSA adsorption of 4.08 μg/cm^2 of P14-Li-2-Si–COOH-2.0 and 2.34 μg/cm^2 of P14-Li-2-Si–COOH–PEG-2 at the stirring speed of 0 rpm
2013[3]	PSPP	PHFBM	60	Oil/water emulsion	60 min, 200, 0.8%	Deionized water surface washing, 99.9%	/

2014[4]	PEO	PDMS	93	BSA	60 min, 200, 19.1%	Deionized water surface washing, 100%	BSA adsorption of 1 μg/cm^2 at the stirring speed of 200 rpm and 18 μg/cm^2 at 0 rpm
2015[5]	PEGMA	PHFBM	90	Oil/water emulsion	60 min, 300, 10.6%	Deionized water backwashing, 99.4%	FDR of 42.3% and FRR of 93.5% at the stirring speed of 0 rpm
2016[6]	PA	HFBM	22	Oil/water emulsion	24 h, 400, 9.2%	Deionized water surface washing, 96.2%	/
2016[7]	PEGMA	PTFOA	90	Oil/water emulsion	60 min, 200, 18.8%	Deionized water surface washing, 99.8%	/
2018[8]	Sulfonic acid groups of PFSA and SiO_2 NPs	Perfluorinated groups of PFSA	269.2	Oil/water emulsion	60 min, 300, 17.4%	Deionized water surface washing, 99.8%	BSA adsorption of 5 μg/cm^2 at the stirring speed of 150 rpm and 15 μg/cm^2 at 0 rpm

2018[9]	PEGMA	PHFBM	90	Oil/water emulsion	60 min, 200, 6.3%	Deionized water surface washing, 99.7%	BSA adsorption of 162.03 μg/cm^2 of PVDF/PEG and 1.41 μg/cm^2 of PVDF/PHFBM-PEGMA at the stirring speed of 150 rpm
2019[10]	PEI	PFOS	220.3	Oil/water emulsion	24 h, 300, 8.3%	Deionized water surface washing, 99.8%	BSA adsorption of 12 μg/cm^2 of HPAN-PEI and 3 μg/cm^2 of HPAN-PEI-PFOS at the stirring speed of 300 rpm
2020[11]	PEI-GO	PFSA	406.7	Vacuum pump oil/water emulsion	60 min, 300, 8.6%	Deionized water surface washing, 98.6%	BSA adsorption of 2 μg/cm^2 at the stirring speed of 150 rpm and 19 μg/cm^2 at 0 rpm
2020[12]	TA	PHFBM	660	Vacuum pump oil/water emulsion	12 h, /, 0	Ethanol/water washing 100%	/
2020[13]	PMEDSAH	PTFEMA	3	SA	6 h, /, 55%	Deionized water surface washing, 74%	Bacterial coverage of 1 % at the stirring speed of 100 rpm and 3 % at 0 rpm

2021[14]	PEGMA	TFEMA	340.46	BSA	60 min, /, 31.91%	Deionized water surface washing, 91.49%	/
2022[15]	PA	PFOC	28.5	Pump oil/water emulsion	8 h, /, 9.5%	Deionized water surface washing, 97.9%	/
2022[16]	PVDF-CTFE membrane modified by itaconic acid	Discontinuous silicon-island structure constructed by SiO2 nanoparticles and PDMS	140	Soybean oil/water emulsion	3h, /, 19.6%	Alkaline solution agitation cleaning, 99.3%	/
2024[17]	PEGMA	PHFBM	325	BSA	2h, /, 58%	Deionized water surface washing, 92.8%	/

2024[18]	TEOA	POTS	6.6	BSA	4h, /, 28.47%	Deionized water surface washing, 99.06%	

PEGMA: Poly(polyethylene glycol methacrylate); PHFBM: Polyhexafl uorobutyl methacrylate; PEG: Polyethylene glycol; PSPP: Poly[3-(Methacryloylamino)propyl]-dimethyl(3-sulfopropyl)ammonium hydroxide; PEO: Poly(ethylene oxide); PDMS: Polydimethylsiloxane; HFBM: 2,2,3,4,4,4-hexauorobutyl methacrylate; TFOA: 3,3,4,4,5,5,6,6,7,7,8,8,8-trideca-fluorooctyl acrylate; PFSA: Perfluorosulfonic acid; PEI: Polyethylenimine; TA: Tannic acid; PMEDSAH: Poly[2-(methacryloyloxy)ethyl-dimethyl-(3-sulfopropyl) ammonium hydroxide]; PTFEMA: Poly(2,2,2-trifluoroethyl methacrylate); TFEMA: Trifluoroethyl methacrylate; PHFB: Poly(hexafluorobutyl methacrylate); TEOA: Triethanolamine; POTS: Perfluorooctyltrimethoxysilane.

Supplementary Table 12 Comparison of reported hydrophilic membrane performances

Report date	Hydrophilic substances - zwitterionic copolymer	Membrane permeability ($Lm^{-2}h^{-1}bar^{-1}$)	Model foulants	Fouling duration, stirring speed (rpm), flux decline rate (FDR)	Cleaning methods, flux recovery rate (FRR)
2007[19]	PEO-g- PAN	159	BSA	24 h, 500, 35%	Deionized water backwashing, 100%
2017[20]	MPC	4.74	Real textile wastewater	6.5 h, cross-flow filtration, 11%	Deionized water surface washing, 100%
2017[20]	MPC	4.74	High concentration mixture of EfOM components	6.5 h, cross-flow filtration, 8%	Deionized water surface washing, 100%
2017[21]	SBMA	5.83	BSA	24 h, /, 13%	Deionized water surface washing, 99%
2018[22]	PAES-co-SBAES	2.5	BSA	12 h, cross-flow filtration, 10%	Deionized water surface washing, 94%
2020[23]	P(TFEMA-OEGMA-AHPMA)	3.1	BSA	40 h, /, 18%	Deionized water surface washing, 99%

2021[24]	DMAPAPS	364	HA	30 min, /, 46%	Deionized water backwashing, 98.1%
2023[25]	MPC/QDMA	255.4	BSA	30 min, /, 65%	Deionized water surface washing, ~100%
2024[26]	SBMA	1000	BSA	2 h, /, 60%	Deionized water surface washing, 88%
2024[27]	MDSA-PEGDA hydrogel	246	BSA	3 h, /, 33.3%	Deionized water surface washing, 80%
2025[28]	P(4VP-co-MPC)	150	BSA	30 min, /, 23.1%	Deionized water surface washing, 95.3%

SBMA: Sulfobetaine methacrylate; MPC: 2-methacryloyloxyethyl phosphorylcholine; PAES-co-SBAES: Poly(arylene ether sulfone-co-sulfobetaine arylene ether sulfone); P(TFEMA-OEGMA-AHPMA): poly(trifluoroethyl methacrylate-co-oligo-(ethylene glycol) methyl ether methacrylate-co-(3-azide-2-hydroxypropyl methacrylate)) ; DMAPAPS : Sulfonated 3-Dimethylaminopropylamine; MPC/QDMA: Methacryloyloxyethyl phosphorylcholine/ 2-(methacryloyloxyethyl)trimethylammonium iodide; MDSA: [2-(Methacryloyloxy) ethyl] dimethyl-(3-sulfopropyl) ammonium hydroxide; PEGDA: Poly (ethylene glycol) diacrylate P(4VP-co-MPC): Poly(4-vinylpyridine-co-methylacryloxyethyl phosphocholine).

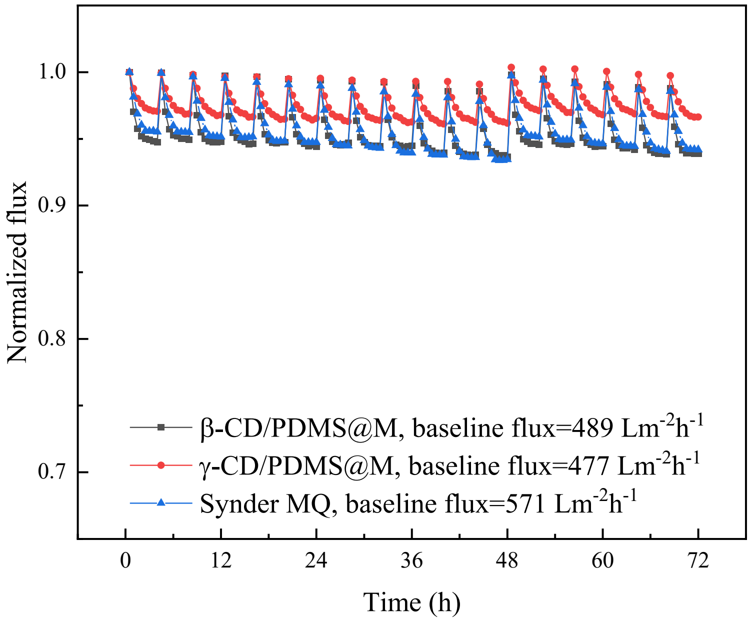

Supplementary Fig. 11 Variations in membrane flux of CD/PDMS@Ms and Synder Filtration MQ membrane with feeds of untreated Songhua River in 72-hour cross-flow ultrafiltration, the baseline flux refers to the stabilized membrane flux after 30 minutes of operation. The $r_{CD:PDMS}$ of β-CD/PDMS@M and γ-CD/PDMS@M were 0.40 and 0.27, respectively. Experiments were conducted at 20 °C with a transmembrane pressure of 1.0 bar and a tangential flow velocity of approximately 0.12 m/s when the membrane flux was 500 $L \cdot m^{-2} \cdot h^{-1}$.

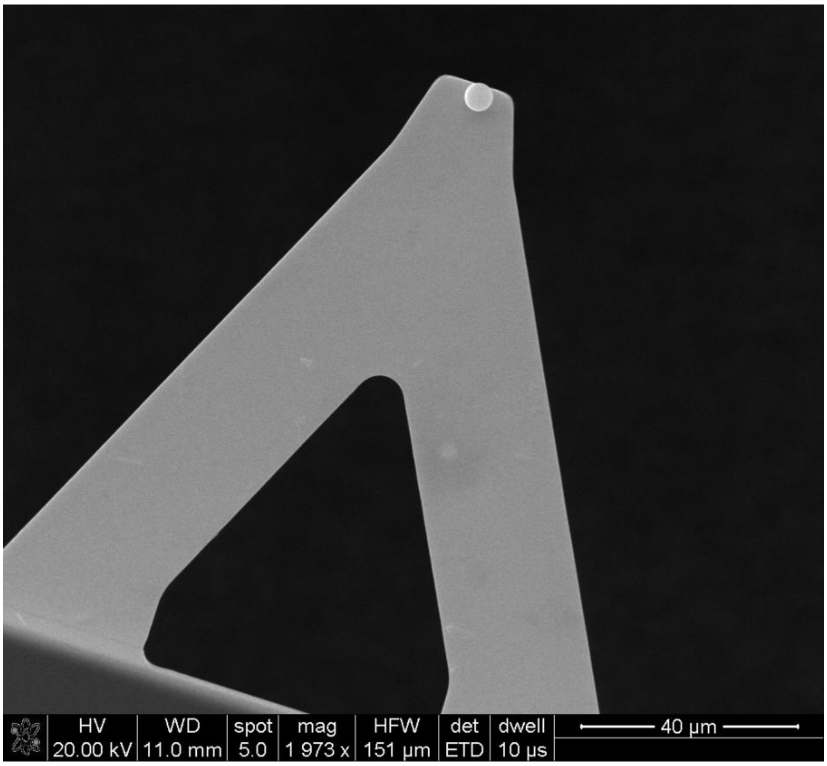

Supplementary Fig. 12 SEM image of probe loaded with BSA-modified SiO_2 microspheres.

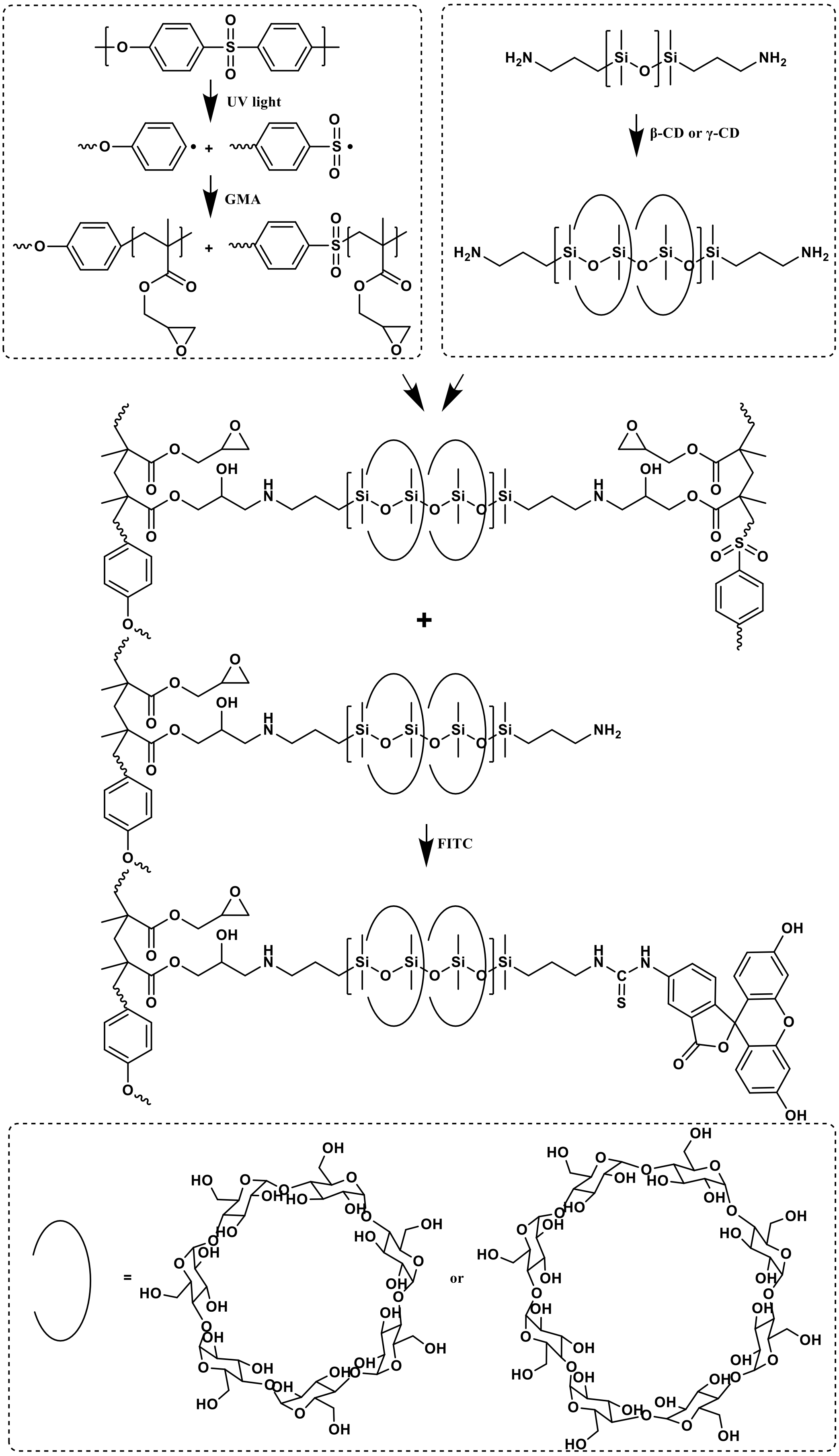

Supplementary Fig. 13 Graphical loading routes of CD/PDMS PPRs on base membrane.

3 Supplementary References

1. Chen W, Su Y, Peng J, Dong Y, Zhao X, Jiang Z. Engineering a robust, versatile amphiphilic membrane surface through forced surface segregation for ultralow flux-decline. *Advanced Functional Materials* **21**, 191-198 (2011).

2. Wu H, Mansouri J, Chen V. Silica nanoparticles as carriers of antifouling ligands for PVDF ultrafiltration membranes. *Journal of Membrane Science* **433**, 135-151 (2013).

3. Zhao X*, et al.* Hierarchically engineered membrane surfaces with superior antifouling and self-cleaning properties. *Journal of Membrane Science* **441**, 93-101 (2013).

4. Zhao X, Su Y, Li Y, Zhang R, Zhao J, Jiang Z. Engineering amphiphilic membrane surfaces based on PEO and PDMS segments for improved antifouling performances. *Journal of Membrane Science* **450**, 111-123 (2014).

5. Liu Y, Su Y, Li Y, Zhao X, Jiang Z. Improved antifouling property of PVDF membranes by incorporating an amphiphilic block-like copolymer for oil/water emulsion separation. *RSC Advances* **5**, 21349-21359 (2015).

6. Zhang R*, et al.* Engineering amphiphilic nanofiltration membrane surfaces with a multi-defense mechanism for improved antifouling performances. *Journal of Materials Chemistry A* **4**, 7892-7902 (2016).

7. Zhang G, Jiang J, Zhang Q, Zhan X, Chen F. Amphiphilic poly(ether sulfone) membranes for oil/water separation: Effect of sequence structure of the modifier. *AIChE Journal* **63**, 739-750 (2016).

8. Zhou L*, et al.* Constructing dual-defense mechanisms on membrane surfaces by synergy of PFSA and SiO2 nanoparticles for persistent antifouling performance. *Applied Surface Science* **440**, 113-124 (2018).

9. Gao K*, et al.* Creation of active-passive integrated mechanisms on membrane surfaces for superior antifouling and antibacterial properties. *Journal of Membrane Science* **548**, 621-631 (2018).

10. Yu Q*, et al.* Assembly of self-cleaning perfluoroalkyl coating on separation membrane surface. *Applied Surface Science* **496**, 143674 (2019).

11. Hao Y, Zhou L, Su Y, Jiang Z. Incorporating dual-defense mechanism with functionalized graphene oxide and perfluorosulfonic acid for anti-fouling membranes. *Separation and Purification Technology* **234**, 116082 (2020).

12. Wang Y, Zhang Y, Wang J. Nano spinel CoFe2O4 deposited diatomite catalytic separation membrane for efficiently cleaning wastewater. *Journal of Membrane Science* **615**, 118559 (2020).

13. Yang Z*, et al.* Antifouling thin-film composite membranes with multi-defense properties by controllably constructing amphiphilic diblock copolymer brush layer. *Journal of Membrane Science* **614**, 118515 (2020).

14. Liu H, Liu Y, Qin Y, Huang Y, Chen K, Xiao C. Amphiphilic surface construction and properties of PVC-g-PPEGMA/PTFEMA graft copolymer membrane. *Applied Surface Science* **545**, 148985 (2021).

15. Li W*, et al.* Perfluorooctanoyl chloride engineering toward high-flux antifouling polyamide nanofilms for desalination. *Journal of Membrane Science* **644**, 120166 (2022).

16. Ma R*, et al.* Constructing discontinuous silicon-island structure with low surface energy based on the responsiveness of hydrophilic layers to improve the anti-fouling property of membranes. *Journal of Membrane Science* **659**, 120770 (2022).

17. Zhang Z*, et al.* Engineering antifouling membrane with surface segregation agents bearing crosslinked low surface energy segments. *Journal of Membrane Science* **691**, 122252 (2024).

18. Chen Y, Wu G, Xue H, Ren Y, Duan X. Engineering nanofiltration membranes based on a two-step modification process with enhanced antifouling and salt separation performance. *Separation and Purification Technology* **330**, 125546 (2024).

19. Asatekin A, Kang S, Elimelech M, Mayes AM. Anti-fouling ultrafiltration membranes containing polyacrylonitrile-graft-poly (ethylene oxide) comb copolymer additives. *Journal of Membrane Science* **298**, 136-146 (2007).

20. Bengani-Lutz P, Zaf RD, Culfaz-Emecen PZ, Asatekin A. Extremely fouling resistant zwitterionic copolymer membranes with ∼ 1 nm pore size for treating municipal, oily and textile wastewater streams. *Journal of Membrane Science* **543**, 184-194 (2017).

21. Bengani-Lutz P, Converse E, Cebe P, Asatekin A. Self-assembling zwitterionic copolymers as membrane selective layers with excellent fouling resistance: Effect of zwitterion chemistry. *Acs Applied Materials & Interfaces* **9**, 20859-20872 (2017).

22. Yang Y, Ramos TL, Heo J, Green MD. Zwitterionic poly(arylene ether sulfone) copolymer/poly(arylene ether sulfone) blends for fouling-resistant desalination membranes. *Journal of Membrane Science* **561**, 69-78 (2018).

23. Hoffman JR, Phillip WA. Dual-functional nanofiltration membranes exhibit multifaceted ion rejection and antifouling performance. *Acs Applied Materials & Interfaces* **12**, 19944-19954 (2020).

24. Cheng K*, et al.* Rapid and robust modification of PVDF ultrafiltration membranes with enhanced permselectivity, antifouling and antibacterial performance. *Separation and Purification Technology* **262**, 118316 (2021).

25. Han DJ, Kim JF, Lee JC, Nam U, Cho YH, Sohn EH. Design of an ionic PVDF-based additive for PVDF water purification membranes with anti-fouling and bactericidal activities. *Journal of Membrane Science* **683**, 121839 (2023).

26. Das P*, et al.* Light-responsive zwitterionic membrane surface modification for antifouling and antibacterial application. *Chemical Engineering Journal* **500**, 157337 (2024).

27. Chen MS*, et al.* Inhibition of biofouling by in-situ grown zwitterionic hydrogel nanolayer on membrane surface in ultralow-pressurized ultrafiltration process. *Water Research* **253**, 121263 (2024).

28. Wang Z-P, Cheng Z, Liu Q, Hou C, An Q-F. Enhanced antifouling PSE hollow fiber membrane via zwitterionic copolymer segregation. *Journal of Membrane Science* **716**, 123512 (2025).
