# Supplementary material for: Supramolecular dynamics-enhanced synergistic antifouling mechanisms for enhanced membrane antifouling and permeability
Source: Nat Commun. 2025 Jul 30;16:6956. doi: 10.1038/s41467-025-62231-w (PMC12311053; doi:10.1038/s41467-025-62231-w)
Supplement: Supplementary file 3 — Description of Additional Supplementary Files [file 41467_2025_62231_MOESM3_ESM.pdf]

Description of Additional Supplementary Files

File name: Supplementary Movie 1

Description: Movement and deformation trajectory of β-CD threaded onto PDMS chains during energy barrier calculations. For clarity, all β-CD atoms are shown in blue.

File name: Supplementary Movie 2

Description: Movement and deformation trajectory of γ-CD threaded onto PDMS chains during energy barrier calculations. For clarity, all γ-CD atoms are shown in blue.
